# Factors influencing the complex problem-solving skills in reflective learning: results from partial least square structural equation modeling and fuzzy set qualitative comparative analysis

**DOI:** 10.1186/s12909-023-04326-w

**Published:** 2023-05-25

**Authors:** Ying Wang, Ze-Ling Xu, Jia-Yao Lou, Ke-Da Chen

**Affiliations:** Shulan International Medical College, Zhejiang Shuren University, Hangzhou, 310015 China

**Keywords:** Reflective learning, Complex problem-solving skills, Partial least squares structural equation modeling, Fuzzy set qualitative comparative analysis

## Abstract

**Background:**

The Organization for Economic Cooperation and Development emphasizes the importance of complex problem-solving (CPS) skills in the 21st century. CPS skills have been linked to academic performance, career development, and job competency training. Reflective learning, which includes journal writing, peer reflection, selfreflection, and group discussion, has been explored to improve critical thinking and problem-solving abilities. The development of various thinking modes and abilities, such as algorithmic thinking, creativity, and empathic concern, all affect problem-solving skills. However, there is a lack of an overall theory to relate variables to each other, which means that different theories need to be integrated to focus on how CPS skills can be effectively trained and improved.

**Methods:**

Data from 136 medical students were analyzed using partial least square structural equation modeling (PLSSEM) and fuzzy set qualitative comparative analysis (fsQCA). A hypothesized model examining the associations between the CPS skills and influence factors was constructed.

**Results:**

The evaluation of the structural model showed that some variables had significant influences on CPS skills, while others did not. After deleting the insignificant pathways, a structural model was built, which showed that mediating effects of empathic concern and critical thinking were observed, while personal distress only had a direct effect on CPS skills. The results of necessity showed that only cooperativity and creativity are necessary conditions for critical thinking. The fsQCA analysis provided clues for each different pathway to the result, with all consistency values being higher than 0.8, and most coverage values being between 0.240 and 0.839. The fsQCA confirmed the validity of the model and provided configurations that enhanced the CPS skills.

**Conclusions:**

This study provides evidence that reflective learning based on multi-dimensional empathy theory and 21 stcentury skills theory can improve CPS skills in medical students. These results have practical implications for learning and suggest that educators should consider incorporating reflective learning strategies that focus on empathy and 21 stcentury skills to enhance CPS skills in their curricula.

**Supplementary Information:**

The online version contains supplementary material available at 10.1186/s12909-023-04326-w.

## Introduction

When putting forward the theoretical framework of skills and competencies in the 21st century, the Organization for Economic Cooperation and Development takes complex problem-solving (CPS) skills as an important component and brings them into the evaluation system of the Program for International Student Assessment [1]. Previous research results have proved that there is a significant positive correlation between CPS skills and academic performance [2], that is, the stronger the problem-solving skill, the better the academic performance. Similarly, it is also considered to have a great influence on career selection [3], career development [4], and job competency training [5]. Therefore, the improvement of the above-mentioned comprehensive qualities, such as learning ability and post competence, and the cultivation of CPS skills, has been emphasized by a variety of teaching strategies, such as problem-based learning (PBL) [6], context-based learning (CBL) [7], situational simulation [8], and reflective learning [9].

As an important process of metacognition, reflective learning is closely related to CPS skills. Gadbury-Amyot et al. claimed that the use of reflection and writing as educational strategies to promote critical thinking and problem-solving is one of the best ways for students to express their thought processes [10]. Exploration to improve CPS skills based on reflective learning and training can be seen in medicine, computer science, mathematics, and other industries. According to Bernack, establishing problem-solving training courses could feasibly enhance the abilities of pre-service teachers [11]. Kellogg suggested that reflection and writing, as educational strategies to promote critical thinking and problem-solving skill, is one of the best ways to improve students’ expression ability and logical thinking [12]. “Reflective learning” is a common way of exploring problems and solutions in the deliberative environment, a process of learning through experience, and is a necessary learning tool in professional education [13]. Reflective learning includes journal writing, peer reflection, self-reflection, and group discussion under the guidance of teachers [14]. Illeris suggested that the result of reflective learning spans cognitive, psychodynamic, and social-societal dimensions [15]. Through its influence on students’ behavior, thoughts, and emotions, it realizes the training and improvement of students’ various abilities. It has gradually developed into a more efficient and autonomous learning model and has become an indispensable educational and learning tool for many professionals [16]. Many experts suggest that the implementation of reflective learning can improve students’ critical thinking [17], insight [18], empathic concern [19], computational thinking [20], and other skills, and this improvement of a variety of thinking modes and abilities will eventually lead to improvement of their CPS skills [21]. Our research on the factors affecting the CPS skills is based on reflective learning.

The purpose of human problem solving is to promote the understanding of human thinking through a detailed investigation of the way people solve difficult problems, such as logic or chess. Unlike computer simulations, human problem solving is influenced by psychological factors that cannot be ignored [22]. Therefore, problem solving is dynamic and needs to consider the influence of speculation, social background, and culture, while CPS skills emphasize the process of successful interaction between the problem solver and the dynamic task environment [23]. CPS skills are collections of self-regulating psychological processes necessary in the face of complex and dynamic non-routine situations across different domains [24], and comprises a combination of skills, abilities, motivation, and other psychological structures [25, 26]. The factors that impact CPS skills are complex and include cognitive and non-cognitive factors. Research shows that the development of a variety of thinking modes and abilities, such as algorithmic thinking, cooperativity, creativity, critical thinking, personal distress, fantasy, perspective-taking, and empathic concern, all affect the problem-solving skill in varying degrees [27, 28]. Among them, empathetic concern and critical thinking have been proven to affect problem-solving skill by many studies. After comprehensively exploring the emerging research on the impact of the factors on the CPS skills, we found that previous studies mainly focused on a single causality in the improvement of problem-solving skill, while there is a lack of overall theory to relate variables to each other, which means that we need to integrate different theories to advance existing research and focuson how CPS skills can be effectively trained and improved.

## Theoretical background

The literature analysis of CPS skills reveals the current research status. Based on the relevant theories of skills needed in the 21st century [1], individuals use analytical, reasoning, and cooperative skills to identify and solve problems consistent with their areas of interest [29]. Kocak proposes that problem-solving skills are shaped by algorithmic thinking, creativity, cooperativity, critical thinking, digital literacy, and effective communication, and develops a model with critical thinking as a mediating factor [21]. Developing solutions for complex problems is a complicated process, and individuals require critical thinking skills [21, 30] to do so. Critical thinking often occurs at the same time as CPS skills and is one of the core objectives of general education in all subjects of higher education [29]. Critical thinking, closely related to reflective learning [17], which has been emphasized in many studies, especially in the implementation of learning strategies including reflective learning. In problem-based learning and case-based learning, instructors encourage learners to use critical reflection to engage with subject matter and to develop their own practice in closing any knowledge gaps that may exist [31]. Additionally, digital literacy involves the ability to assess the accuracy and value of online resources [32]. In this study, reflective learning was the primary learning strategy [33]; therefore, digital literacy skills were not observed in detail. Drawing on the above analysis, we developed a theoretical model that identifies algorithmic thinking, creativity, and cooperativity as antecedents, and critical thinking as an intermediary variable that influences CPS skills.

Another major area related to affecting CPS skills is empathic concern. The research suggests that students with a higher level of cognitive empathy show more positive attitudes and deal with problems more effectively [34]. In essence, empathetic concern fosters values, beliefs, attitudes, and assumptions, and affect the CPS skills from the perspective of execution [35, 36]. Some studies suggest that reflective learning improves empathy [37]. Based on Davis’s Interpersonal Reactivity Index [38], empathy was divided into four dimensions mentioned: Empathic concern, fantasy, perspective-taking, and personal distress. Nevertheless, some scholars disagree that personal distress belongs to the category of empathy. Personal distress is defined as an over-arousal caused by the lack of boundaries between oneself and others [39, 40]. Some studies show that personal distress leads to egoism and overwhelms altruistic activities mediated by empathetic concern [41]. And there is a statistically significant correlation between personal distress and empathetic concern [42]. Therefore, we still adhere to the view that the two cannot be regarded as mutually exclusive emotions, bringing personal distress into the scope of our research and exploring its role in CPS skills. Empathetic concern has been proved to associate with prosocial behavior [43]. In the relationship between empathic concern and prosocial concern, empathic concern elicits an approach orientation toward the target [44] and is used as a mediator variable in some models. For example, some studies consider empathic concern and personal distress are both mediators of perspective-taking to helping behavior [45]. Based on the above analysis, we built our theoretical model and assume that personal distress, perspective-taking, and fantasy as antecedents and empathetic concern as intermediary variables that affect the CPS skills.

Above all, the empathic concern and the critical thinking are two remarkable characteristics of the CPS skills, which can play a common role in the CPS skills [46], however, there is a lack of overall theory to connect them, which means that different theories need to be integrated to promote research. The comprehensive study of the combination of the two aspects can better understand how to improve CPS skills, which cannot be provided by any theory alone. Moreover, the results on the factors affecting the CPS skills also show some inconsistencies. For example, Batson believes that personal distress in empathy inhibits the development of problem-solving skills [41], whereas Mora disagrees [47]. A possible reasonable explanation for these contradictory results is that the previous studies on the factors influencing problem-solving skill mainly adopted traditional symmetric methods (such as regression and SEM), which did not fully capture the complexity of the factors that influence problem-solving skills, and the factors affecting the CPS skills are often based on multiple causalities rather than a single causal relationship. Simply evaluating symmetric relationships might lead to divergent results, thus masking the complexity of the problem-solving skill. Considering the complex nature of CPS skills under the condition of reflective learning, it is necessary to check the symmetric and asymmetric relationships between structures to fully understand the strategies and methods to improve CPS skills, therefore, PLS-SEM [48] and fsQCA [49] were used in our study comprehensively.

## Research model and hypothesis development

### Designing the PLS-SEM research model

**Fig. 1:**
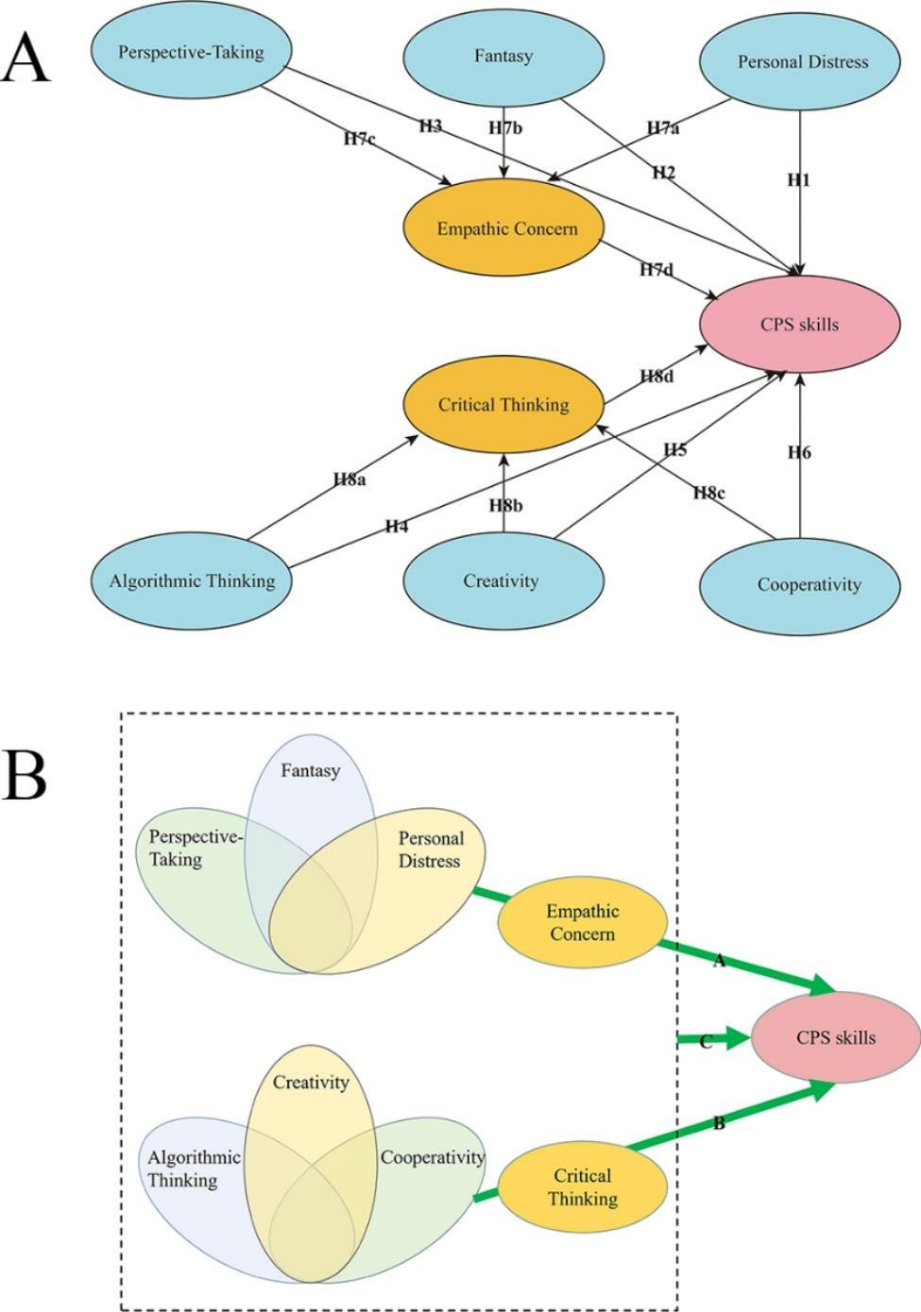
Partial least square structural equation modeling (PLS-SEM) conceptual model and fuzzy set qualitative comparative analysis (fsQCA) conceptual model: (**A**) The PLS-SEM conceptual model. (**B**) The fsQCA conceptual model

Critical thinking in the field of cognition and empathic concern in the field of emotion are representatives of two different thinking modes affecting the CPS skills. PLS-SEM assumes that fantasy, perspective-taking, personal distress, algorithmic thinking, creativity, and cooperativity have a direct impact on the CPS skills. Empathetic concern and critical thinking play an intermediary role between these relationships and the CPS skills (Fig. 1A).

#### Personal distress and CPS skills

The definition of personal distress in this study pertains to the discomfort and anxiety that respondents experience when observing negative experiences of others, including fear, apprehension, and discomfort. Personal distress is an aspect of emotional empathy [38]. Some studies show that personal distress and empathy are complex and dynamic emotional experiences [50]. Personal distress, as an indicator of self-other differentiation and emotional regulation, is a kind of negative emotion. Excessive personal distress can lead to emotional regulation and interpersonal difficulties [51]. These studies advocate reducing personal distress to relieve stress [52]. The healthcare sector prioritizes a patient-centered healthcare model, which mandates that we respond to patients’ emotional distress with this principle in mind. However, in practice, health professionals tend to regard emotional health problems as “routine”; therefore, it is necessary to put patients’ emotional and identity issues in the dominant position of the marginal biomedical model used by health professionals [53]. However, empathic pain is crucial to Hoffman’s moral development framework. He believed that pain can cause significant effects that might lead to action [54]. Moitra’s research also supports the positive effect of personal distress on problem-solving skill [47]. Reflection encourages individuals to confront their own embarrassing and uncomfortable past experiences, learn from their errors, and enhance their CPS skills [55].

#### Fantasy and CPS skills

Fantasy acts on all aspects of reflective learning. First, to some extent, our brains process information and decisions in an irrational way, and reflection contributes to the cultivation of irrational thinking [56]. The improvement of subjects’ irrational thinking, including fantasy, can be promoted through reflective learning. Research indicates that individuals with higher fantasy and perspective-taking skills tend to have stronger social understanding [57]. Second, the development of imagination and fantasy is an important part of cultivating empathic concern [58]. This is because people understand the world through fantasy, and fantasy gives people hope that the world will become a better place [59]. For example, Melissa McInnis Brown’s research showed that children who play using fantasies are better at sharing emotions than their peers [60]. Many studies have proven the role of fantasy in problem-solving. For example, David Weibel pointed out that one can effectively use imagination in an environment, such as in artistic expression or problem-solving [61]. Fantasy is an imaginative way to find creative solutions that can help people predict the realization of creative structures [61]. From a sociological point of view, scholars usually regard fantasy as an important factor in cultivating children’s prosocial behaviour [57, 62]. Empathic concern requires a person or the whole team to have an overall and largely unconscious “feeling” in terms of emotions, body language, previous experiences, and interpersonal relationships; therefore, this requires significant support from the fantasy system [63].

#### Perspective-taking and CPS skills

The effectiveness of group problem solving heavily depends on group member interactions and group composition. For perspective-taking, it provides the possibility for effective communication, which mainly affects the effective presentation of information, effective understanding of that information, conflict resolution, and cooperative interaction [64, 65]. In management, perspective-taking has become an important factor in teamwork to solve problems [66]. During perspective-taking, the problem-solving process can be facilitated by promoting empathic concern, which is evident in the subjects’ cognitive dimension. For example, Falk found that perspective-taking leads to more creative solutions, and team members are more cooperative and facilitate more effective communication [64]. Bethune and Brown suggested that reflection affects the professional identity of patients by encouraging personal insights and providing different perspectives on patient interaction [67]. Reflection requires us to think about the past and sum up experiences and lessons from it. Thinking about problems from the standpoint of others can circumvent the limitations of our perspective of looking at problems only through ourselves and can promote the solution of complex problems.

Based on the points discussed above, we propose the following assumptions:

##### H1

Personal distress is positively related to the CPS skills.

##### H2

Fantasy is positively related to the CPS skills.

##### H3

Perspective-taking is positively related to the CPS skills.

#### Algorithmic thinking and CPS skills

Algorithm thinking draws lessons from the algorithms of computers and artificial intelligence, which enables people to think and deal with things in parallel, process things in data, carry on data and logical reasoning to things, and finally achieve the goal of completing plans and tasks. As one of the core skills in the 21st century, algorithmic thinking abstractly and logically determines the elements used to solve problems through analysis [28]. One of the major applications of algorithmic thinking is jigsaw puzzle-based learning, which aims to make subjects think about how to build and solve problems, and improve their critical analysis and problem-solving skills [68]. Hasan Gürbüz leveraged straightforward visual and language templates to help individuals develop models and analyze information about events through games, resulting in improved problem-solving skills [69]. This mode of thinking, based on logic and steps, is very important for the development of critical thinking and computational thinking [28]. Many studies have shown that there is a positive correlation between algorithmic thinking and critical thinking [70]. In reflective learning, algorithmic thinking plays a significant role in computing, as evidenced in this study by recording a short video that necessitates organizing large amounts of data to develop suitable algorithms for analysis [71].

#### Creativity and CPS skills

Creativity affects our lives and is vital to the progress of society [72]. The definition of creativity highlights the integration of novel (original, unexpected) and appropriate (useful, adaptive concerning task constraint) ideas [73]. Since the 20th century, a large number of scholars in various fields have paid attention to creativity and CPS skills. Creativity is a valuable skill while designing solutions to new challenges that arise in developing societies [74]. For instance, Garrett noted that creativity plays a crucial role in problem-solving [75]. In many studies, creativity and critical thinking are interdependent, and creative tasks can improve people’s creativity [76]. In reflective learning, we utilize divergent thinking that frequently enhances our creativity.

#### Cooperativity and CPS skills

Many critics believe that cooperativity plays an important role in the cultivation of critical thinking [77]. Cooperativity receives considerable attention in the learning process due to its association with effective communication. For example, service-learning attaches great importance to cooperation, democratic citizenship, and moral responsibility in the learning process [78], and preschool educational institutions need to improve the experience through the collaborative exchange, to create favorable conditions for educators to re-examine educational activities, and determine the direction of new relationships through observation [79]. In reflective learning, subjects become aware of their contradictions and gain valid information, and critically assess peer opinions through active communication, which advances their ideas for program and CPS skills improvement.

Based on the points discussed above, we propose the following assumptions:

##### H4

Algorithmic thinking is positively related to the CPS skills.

##### H5

Creativity is positively related to the CPS skills.

##### H6

Cooperativity is positively related to the CPS skills.

#### Mediators and CPS skills

This study assumes that empathetic concern and critical thinking act as mediators between the CPS skills and their antecedents.

In Gibbs’s theory, the emotional dimension is a very important aspect of reflective learning [80]. Madeline Kelly’s research showed that reflection has a positive effect on the improvement of cognitive empathy [81]; however, there are few studies on the effect of reflective learning on empathy. Cognitive empathy includes fantasy and perspective-taking, while the emotional empathy includes personal distress and empathic concern [82]. Research shows that the concept of emotional empathy is state empathy, with the focus on altruism [83, 84]. Emotional empathy plays an important role in patient-nurse communication [85]. Failure to deal with or understand emotions will make it difficult for nurses to think rationally and critically about issues that are important to nursing practice [86]. Therefore, we cannot ignore the influence of empathic concern on the CPS skills in reflective learning. We assumed that the ability of empathic concern can increase altruism and help to improve CPS skills. However, personal distress is usually considered to lead to egoism, which is not conducive to the formation of altruism [41]. In-depth investigation is necessary to understand its effect on CPS skills. As an important factor in prosocial behavior, the empathic concern serving as a mediator between cognitive behavior and prosocial behavior [87]. Based on the theories of O’Brien and Gülseven, we constructed a CPS skills model with empathic concern as the mediating variable [88, 89].

Effective reflection is characterized by purposeful, focused, and questioning [90]. In the process of reflection, this mode of thinking requires us to think critically and center on the results. Reflective learning, also known as critical reflection [17], emphasizes the use of critical thinking. Many critics affirm the results of critical reflection [91–93]. Parrish and Crookes found that among nursing graduates, reflection helped them to solve problems through thoughtful reasoning and to develop strategies for self-monitoring of their professional competence [94]. Critical thinking is typically rational thinking, and through combining theory with practice, exploring the similarities and differences between theoretical knowledge and practical experience, and considering a variety of different viewpoints and opinions, the effect of reflective learning can be enhanced. Therefore, speculative reflection is designed to help us identify our shortcomings and think about how to correct and improve them. Critical thinking is widely recognized as an important skill in mediating CPS skills [10]. Based on the research of Kocak and Tee, we also view critical thinking as an intermediary variable, playing a mediating role in algorithmic thinking, creativity, and cooperativity within CPS skills [21, 95].

Based on the points discussed above, we propose the following assumptions:

##### H7a

Personal distress indirectly affects the CPS skills through empathic concern.

##### H7b

Fantasy indirectly affects the CPS skills through empathic concern.

##### H7c

Perspective-taking indirectly affects the CPS skills through empathic concern.

##### H7d

Empathic concern is positively related to the CPS skills.

##### H8a

Algorithmic thinking indirectly affects the CPS skills through critical thinking.

##### H8b

Creativity indirectly affects the CPS skills through critical thinking.

##### H8c

Cooperativity indirectly affects the CPS skills through critical thinking.

##### H8d

Critical thinking is positively related to the CPS skills.

### Designing the fsQCA configuration model

In this study, a Venn diagram is used to design the fsQCA configuration model (Fig. 1B), which was used to explore the causal model for improving CPS skills. In the diagram, arrow A represents a combination of perspective-taking, fantasy, and personal distress, and adds configurations that affect the CPS skills through, or including, empathetic concern. Arrow B represents a combination of algorithmic thinking, creativity, and cooperativity, and adds configurations that affect the CPS skills through, or including, critical thinking. Arrow C represents the combination of all the variables and represents the complex interaction of these factors to predict the resulting conditions.

## Methods

### Participants

Participants were 136 freshmen and medical majors from a university in southeastern China (‾Xage = 18.47, female = 82.35%, male = 17.65%). The inclusion criterion comprised students who had conducted reflective learning. The exclusion criteria comprised: (1) Students who did not make reflective videos, or (2) students suspected of plagiarizing reflective learning achievements. A total of 163 cases were included in the empirical study of reflective learning, and 136 effective samples were recovered, with an effective recovery rate of 83.44%.

### Design and procedure

**Fig. 2:**
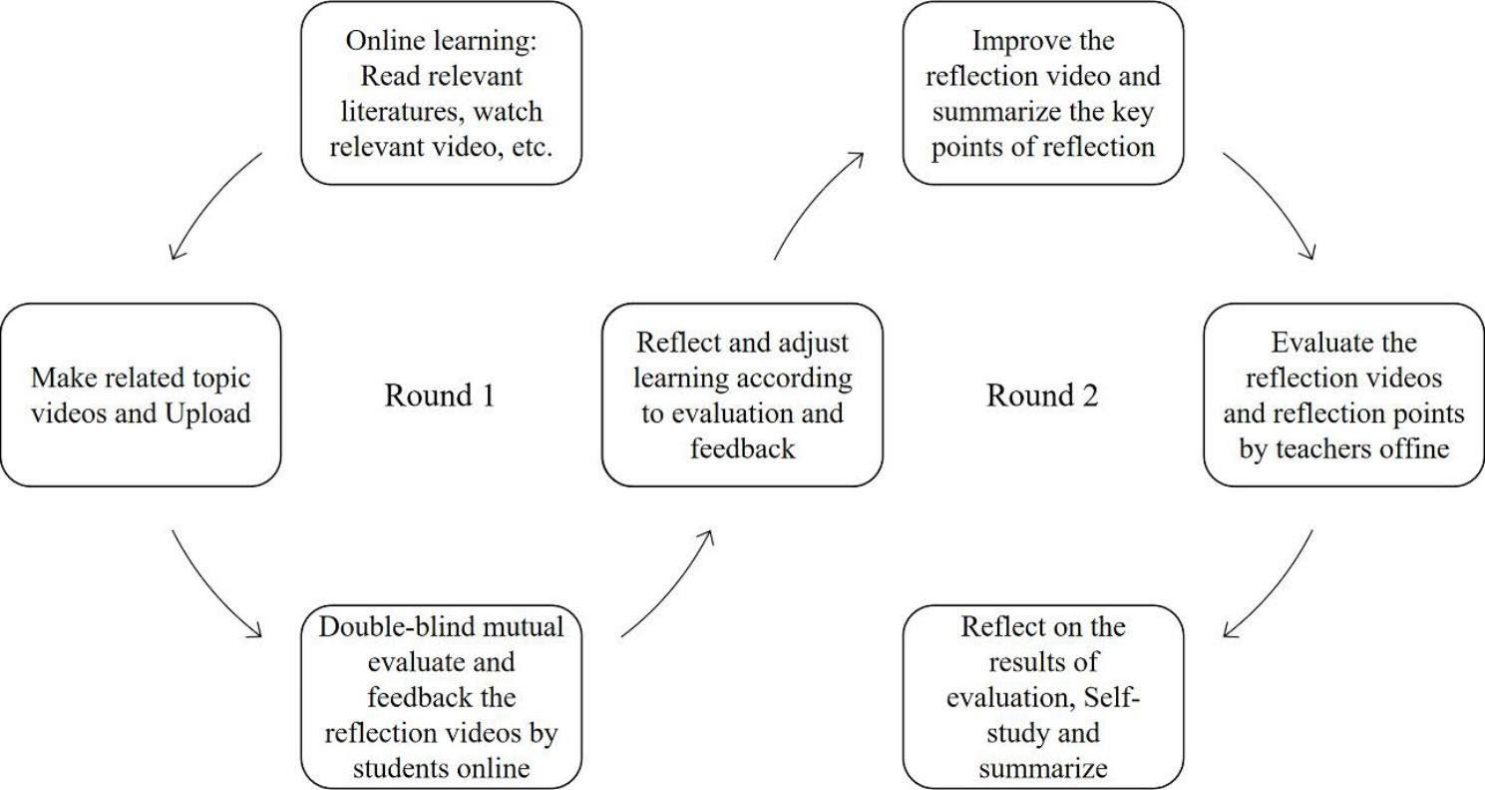
Reflective learning process

After receiving appropriate online training, classroom teachers implemented a reflective learning curriculum design among medical students in the autumn of 2021 (Fig. 2). Based on the *Biochemistry and Molecular Biology* Courses, the two rounds of teaching plan lasted a total of 14 weeks was design. In the first round of reflective learning, subjects were asked to read relevant literature, watch relevant video materials, etc., and carry out online learning. They were then asked to record learning videos on their own, and then upload the videos, followed by a double-blind mutual evaluation of learning videos between online students. In the second round of reflective learning, students adjusted their reflective learning according to the feedback from the previous round of mutual evaluation, implemented a second round of deeper material learning exploration, improved their reflective video, and summarized the main points of reflective learning. Teachers evaluated the reflective videos and learning points offline, and students learned and summarized according to the evaluation results. After the end of the entire process, we issued a competency assessment questionnaire to measure learners’ competency levels and the data was collected.

### Instrument

To measure the constructs under study, existing scales were used (see Table 1 for items associated with each construct and scale reliabilities).

**Table 1:** The Constructed Items Measurement Model

Scale constructs	Outer loadings
Fantasy (CR = 0.857, CA = 0.749, AVE = 0.669) ^a^	
When I am reading an interesting story or novel, I imagine how I would feel if the events in the story were happening to me (F1)	0.678
I get really involved with the feelings of the characters in a novel (F2)	0.908
After seeing a play or movie, I have felt as though I were one of the characters (F3)	0.851
Perspective-taking (CR = 0.815, CA = 0.571, AVE = 0.669) ^a^	
*If I am sure I am right about something, I do not waste much time listening to other people’s arguments (PT1)	0.741
*I sometimes find it difficult to see things from the “other guy’s” point of view (PT2)	0.912
Personal distress (CR = 0.814, CA = 0.774, AVE = 0.603) ^a^	
In emergency situations, I feel apprehensive and ill-at-ease (PD1)	0.758
Being in a tense emotional situation scares me (PD2)	0.727
I tend to lose control during emergencies (PD3)	0.966
Empathic concern (CR = 0.912, CA = 0.855, AVE = 0.777) ^a^	
*When I see someone being treated unfairly, I sometimes do not feel very much pity for them (EC1)	0.851
*Usually I am not extremely concerned when I see someone else in trouble. (EC2)	0.937
*Sometimes I do not feel sorry for other people when they are having problems. (EC3)	0.854
Algorithmic Thinking (CR = 0.949, CA = 0.919, AVE = 0.860) ^b^	
I believe that I can easily catch the relation between the figures (AT1)	0.898
I can express mathematically the solutions to the problems I face in the daily life (AT2)	0.950
I can digitize a mathematical problem expressed verbally (AT3)	0.934
Creativity (CR = 0.871, CA = 0.777, AVE = 0.693) ^b^	
I like the people who are sure of most of their decisions (C1)	0.768
I believe that I can solve the problems that possibly occur when I encounter a new situation (C2)	0.950
I trust my intuitions and feelings of “trueness” and “wrongness” when I approach the solution to a problem (C3)	0.823
Cooperativity (CR = 0.929, CA = 0.890, AVE = 0.815) ^b^	
I like experiencing cooperative learning together with my group of friends (CA1)	0.871
I like solving problems related to a group project together with my friends in cooperative learning (CA2)	0.931
More ideas occur in cooperative learning (CA3)	0.905
Critical Thinking (CR = 0.924, CA = 0.877, AVE = 0.803) ^b^	
I am good at preparing regular plans regarding the solution of complex problems (CT1)	0.893
I am proud of being able to think with great precision (CT2)	0.880
I make use of a systematic method while comparing the options at hand and while reaching a decision (CT3)	0.915
Problem Solving (CR = 0.921, CA = 0.871, AVE = 0.795) ^b^	
*I have problems in the issue of where and how I should use the variables such as X and Y in the solution of a problem (PS1)	0.893
*I cannot produce many options while thinking of the possible solutions to a problem (PS2)	0.928
*I cannot develop my own ideas in the environment of cooperative learning (PS3)	0.853

(a) Adapted from IRI (1980). (b) Adopted from CTS (2017)

**Note:** PD = Personal Distress, F = Fantasy, PT = Perspective-Taking, EC = Empathic Concern, AT = Algorithmic Thinking, C = Creativity, CA = Cooperativity, CT = Critical Thinking, PS = CPS skills

A questionnaire was developed based on the existing mature scale, and the items were slightly adjusted according to the model. The relationship between the retained items and the dimensions was not complementary. Improvement of CPS skills is described as a structure composed of six antecedent variables and two mediating variables with different ways of thinking. The Davis Interpersonal Reactivity Index (IRI) was used for personal distress, fantasy, perspective-taking, and empathic concern [38], and the Computational Thinking Scale (CTS) was used for critical thinking, algorithmic thinking, creativity, problem solving, and cooperativity [74, 96, 97]. We structured it as personal distress (three items), fantasy (three items), and perspective-taking (two items) as ante-dependent variables, and the mediating effect of empathic concern (three items) on CPS skills (three items) was directly and through empathic concern (3 items). Similarly, algorithmic thinking (3 items), creativity (three items), and cooperativity (three items) acted as ante-variables, both directly and through the mediating effect of critical thinking (three items). All items were evaluated using a Likert 5-point scale, 5 = strongly agree, 4 = agree, 3 = neither agree nor disagree, 2 = disagree, 1 = strongly disagree, and the scores of items in reverse scoring were reversed. Entries for reverse scoring are marked with * in Table 1. The questionnaire was translated into Chinese and distributed after discussion with experts.

### Data analysis

#### PLS-SEM

We use multiple methods to analyze the data. First, PLS-SEM was carried out on the data through Smart-PLS 3.0 software to adapt the complex model analysis and explore the impact of various factors [48].

We measured the characteristics of the structure using internal consistency reliability, convergence validity, and discrimination validity. Internal consistency reliability was measured using the alpha and combinatorial reliability of Cronbach. And we checked the collinearity of the internal model and evaluated the deviation of the method using a variance inflation factor (VIF). According to the research objectives, we tested two models with different paths with significant correlations. The direct predictive effects of fantasy, personal distress, perspective-taking, creativity, cooperativity, and algorithmic thinking, as well as the mediating effects of empathic concern and critical thinking, on CPS skills were tested. A nonparametric, bias-corrected bootstrap with 5,000 subsamples and a 95% confidence interval was used. The structural model was evaluated by R² and by the significance of the estimated value of pathway relationships. The significance of pathway coefficients was evaluated using the bootstrap subsamples, and the structural model was evaluated using 5000 bootstrap subsamples [98]. R² values of 0.25, 0.50, or 0.75 are considered weak, moderate, and significant, respectively.

#### FsQCA

Although PLS-SEM can handle both external (measurement) and internal (structural) models [98], it is limited by symmetry. Therefore, we used fsQCA 3.1 software [49] to analyze asymmetry and obtain a sufficient causal combination configuration to study the complex relationship between variables more comprehensively and in detail. According to the fsQCA user guide, data calibration, truth table construction, and causal condition analysis are necessary steps in the process of data analysis [49]. In the first step, we converted the ordinary data into fuzzy sets by setting the original values from the Likert scale, which corresponded to full membership, cross-over anchors, and full non-membership based on Kallmuenzer’s analysis [99]. The second step is to construct the truth table and generate different combinations of causal conditions that are sufficient to affect the CPS skills by specifying a consistent cutoff value as the natural breakpoint in the consistency and the case number threshold as 1. The third, we analyzed the necessity of all the variables (critical thinking, creativity, algorithmic thinking, cooperativity, empathetic concern, perspective-taking, personal distress and fantasy) to the CPS skills, and the antecedent variables for mediate variables (critical thinking and empathic concern), and the necessity of mediating variables to the outcome variables. It is generally believed that a condition or combination of conditions is “necessary” or “almost always necessary” when the consistency score is higher than 0.9 [49]. Finally, we use standard analysis to obtain “intermediate solutions” (i.e., partial logical remainders are incorporated into the solution) to identify causal patterns that affect CPS skills.

## Results

### The result of PLS-SEM

#### Evaluation of the reflection measurement model

Except for the perspective-taking, the Cronbach’s alpha in the other dimensions was generally more than 0.7, reaching the standard recommended by Cohen (Table 1) [100]. After examining the external loads in the external model, we observed that most of the loads were more than 0.7, while the PD1 project was still less than 0.7. After checking the Cronbach’s alpha and average variance extracted (AVE), we confirmed that this factor had no negative effect on our research [98], and was thus retained the project. The sample size of the model is small (less than 300), and the items considered by perspective-taking are 2 (less than 3), so Cronbach’s alpha is easily less than 0.6. The alpha of perspective-taking is more than 0.5, which is still in a slightly plausible range. Therefore, we kept the item of perspective-taking. Secondly, the square root of AVE was greater than 0.5, which accords with the convergence validity [101]. In addition, we used the Fornell-Larker criteria to evaluate the discriminant validity (Table 2).

**Table 2:** Discriminant Validity using Fornell-Larcker criterion

	Algorithmic Thinking	Cooperativity	Creativity	Critical Thinking	Empathic Concern	Fantasy	Personal Distress	Perspective-Taking	CPS skills
Algorithmic Thinking	0.927								
Cooperativity	0.542	0.903							
Creativity	0.510	0.502	0.832						
Critical Thinking	0.674	0.617	0.672	0.896					
Empathic Concern	0.034	0.165	0.118	0.103	0.881				
Fantasy	0.181	0.242	0.301	0.266	0.243	0.818			
Personal Distress	0.235	0.196	0.096	0.135	0.336	-0.022	0.777		
Perspective-Taking	0.069	0.191	0.117	0.031	0.598	0.117	0.353	0.831	
CPS skills	0.157	0.228	0.213	0.244	0.632	0.234	0.390	0.727	0.892

#### Evaluation of formative measurement models

The results showed that the VIF of all constructs was lower than the threshold of 3.3 (see Additional file. 1) [98]. In order to further analyze, this study evaluated the quality by blindfolding program (Q^2^) and standardized root mean square residual (SRMR). The results showed that SRMR = 0.079, not exceeding 0.09 [102]. The blindfold program showed that Q^2^ was greater than 0, which verified the predictive correlation of the research model [103].

#### Structural model evaluation

**Fig. 3:**
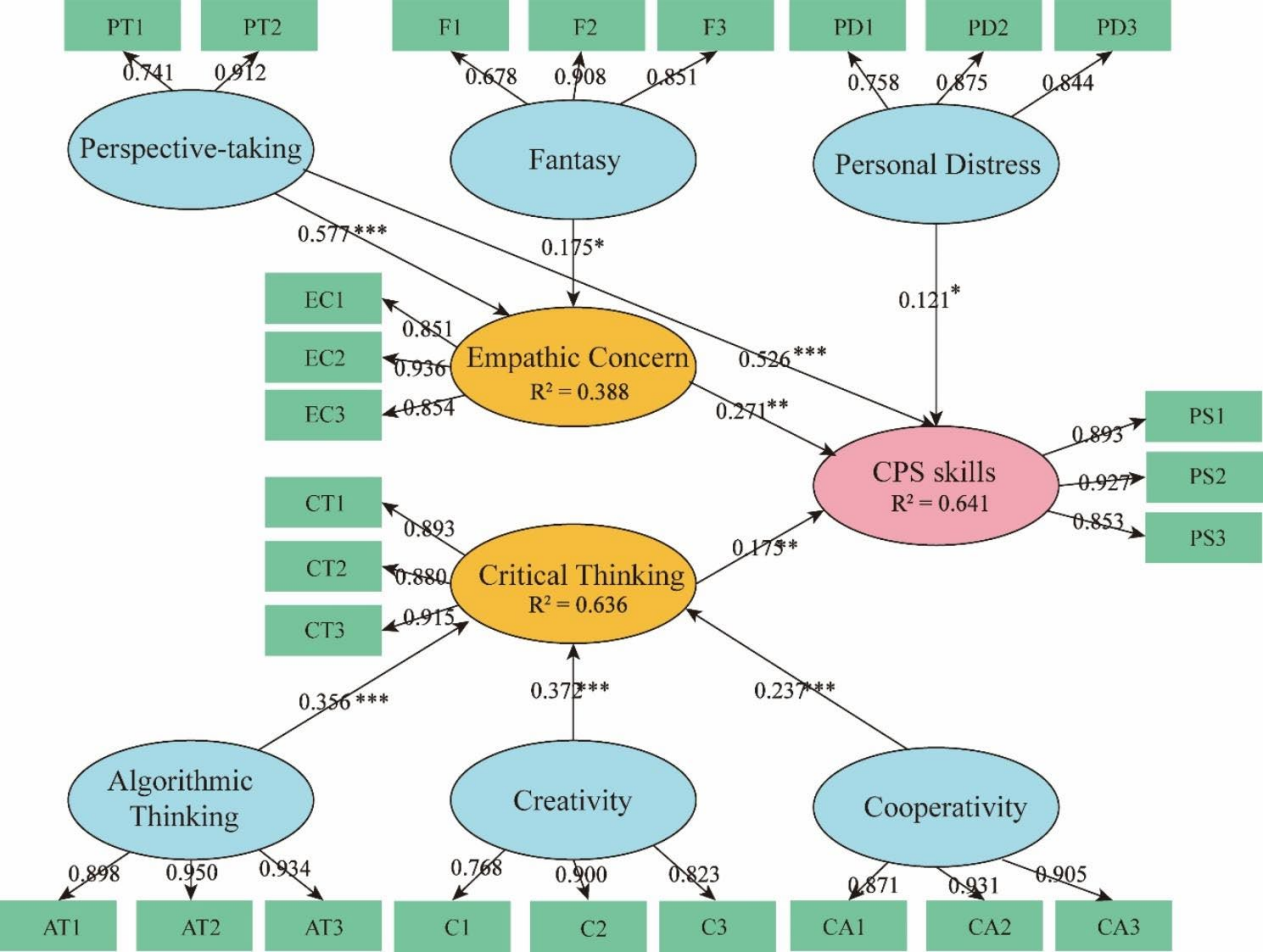
Path model and partial least square structural equation modeling (PLS-SEM) estimates

Evaluation of the structural model showed that the R² value was reasonable for exploratory research. Meanwhile, the direct pathway effect of fantasy, algorithmic thinking, creativity, and cooperativity on CPS skills was not significant (*p* > 0.05), and the pathway effect of personal distress on empathic concern was also not significant (*p* > 0.05). The other variables showed significant influences on CPS skills (*p* < 0.05) (Table 3). After deleting the insignificant pathways, we built a structural model between the CPS skills and the influencing factors (critical thinking, cooperativity, creativity, algorithmic thinking, empathic concern, fantasy, perspective-taking, and personal distress) (Fig. 3). Compared with the hypothetical model, mediating effects of empathic concern and critical thinking were observed; however, personal distress only had a direct effect on CPS skills, which was consistent with the previous view that empathic concern and personal distress should be discussed [51].

**Table 3:** Results of hypotheses testing

Hypothesized path	*t* value	*p* value	Conclusion
Personal Distress → CPS skills	2.063	0.039	H1 is supported
Perspective-Taking → CPS skills	8.252	0.000	H3 is supported
Fantasy → Empathic Concern	2.243	0.025	H7b is supported
Perspective-Taking → Empathic Concern	6.603	0.000	H7c is supported
Empathic Concern → CPS skills	4.194	0.000	H7d is supported
Algorithmic Thinking → Critical Thinking	5.897	0.000	H8a is supported
Creativity → Critical Thinking	5.287	0.000	H8b is supported
Cooperativity → Critical Thinking	3.468	0.001	H8c is supported
Critical Thinking → CPS skills	3.141	0.002	H8d is supported

### The result of fsQCA

The results of necessity showed that only cooperativity and creativity are necessary conditions for critical thinking (see Additional file. 2, Additional file. 3, and Additional file. 4).

FsQCA assessed the complex causal combination that led to improved CPS skills (Tables 4, 5, 6 and 7). The solution provided clues for each different pathway to the result, with all consistency values being higher than 0.8, and most coverage values being between 0.240 and 0.839 [104].

**Table 4:** Configurations for CPS skills

	Raw Coverage	Unique Coverage	Consistency
Causal models from a high level of CPS skills PS = f(F*PT*PD*EC*AT*C*CA*CT)			
C1 = CT*CA*AT*C*EC*PD*PT	0.354	0.025	0.974
C2 = CT*CA*AT*C*EC*PD*F	0.352	0.037	0.950
C3 = CT*CA*AT*C*PD*PT*F	0.340	0.024	0.950
C4 = ~ CT*~CA*~AT*~C*EC*PD*PT*~F	0.240	0.054	0.957
C5 = ~ CT*CA*AT*C*EC*~PD*PT*F	0.243	0.038	0.983
C6 = ~ CT*CA*~AT*C*EC*PD*PT*F	0.250	0.015	0.972
Solution coverage: 0.549667 Solution consistency: 0.9381			
Causal models from a low level of CPS skills ~PS = f(F*PT*PD*EC*AT*C*CA*CT)			
NC1 = ~ CT*~CA*~AT*~C*~EC*~PD*~PT	0.361	0.076	0.972
NC2 = ~ CT*~AT*~C*~EC*~PD*~PT*F	0.312	0.012	0.975
NC3 = ~ CT*~CA*~AT*~C*~PD*~PT*F	0.306	0.028	0.975
NC4 = ~ CT*CA*AT*C*~EC*~PD*PT*~F	0.228	0.031	0.951
NC5 = CT*CA*AT*C*~EC*~PD*~PT*F	0.255	0.067	0.967
Solution coverage: 0.526662 Solution consistency: 0.953452			

**Table 5:** Configurations for Empathic concern

	Raw Coverage	Unique Coverage	Consistency
Causal models from a high level of empathic concern EC = f(F*PT*PD)			
C1 = PT*F	0.612	0.612	0.821
Solution coverage: 0.611858 Solution consistency: 0.821294			
Causal models from a low level of empathic concern ~EC = f(F*PT*PD)			
NC1 = ~ PT*PD	0.567	0.127	0.849
NC2 = ~ F*PT	0.556	0.116	0.889
Solution coverage: 0.682991 Solution consistency: 0.841682			

**Table 6:** Configurations for Critical Thinking

	Raw Coverage	Unique Coverage	Consistency
Causal models from a high level of critical thinking CT = f(AT*C*CA)			
C1 = C*AT*CA	0.760	0.760	0.867
Solution coverage: 0.760238 Solution consistency: 0.869689			
Causal models from a low level of critical thinking ~CT = f(AT*C*CA)			
NC1 = ~ AT	0.766	0.331	0.853
NC2 = ~ CA*C	0.508	0.073	0.961
Solution coverage: 0.839477 Solution consistency: 0.857027			

**Table 7:** Configurations for CPS skills

	Raw Coverage	Unique Coverage	Consistency
Causal models from a high level of CPS skills PS = f(EC*CT)			
C1 = EC*CT	0.550	0.550	0.890
Solution coverage: 0.550111 Solution consistency: 0.890487			
Causal models from a low level of CPS skills ~PS = f(EC*CT)			
NC1 = ~ EC* ~CT	0.656	0.656	0.840
Solution coverage: 0.656391 Solution consistency: 0.839813			

As shown in Table 4, there are six approaches to the final model of complex conditions that lead to high CPS skills, among which the top three in terms of coverage are: (1) To achieve high CPS skills through high critical thinking, cooperativity, creativity, algorithmic thinking, empathic concern, personal distress, and perspective-taking (consistency = 0.974, coverage = 0.354). (2) Under conditions of high critical thinking, cooperativity, algorithmic thinking, and creativity, combined with high empathic concern, personal distress, and fantasy, the CPS skills can be improved (consistency = 0.950, coverage = 0.352). (3) A high level of critical thinking, cooperativity, algorithmic thinking, creativity, personal distress, perspective-taking, and fantasy (consistency = 0.950, coverage = 0.340) can promote the improvement of CPS skills.

To examine the mediating effect of empathic concern and critical thinking on the CPS skill, we analyzed the complex causality of fantasy, personal distress, perspective-taking, and empathic concern. The results showed in Table 5 indicated that the complex causal statement of fantasy, personal distress, perspective-taking, and empathic concern is one way, i.e., high perspective-taking and fantasy improves empathic concern skill (consistency = 0.821; coverage = 0.612), which supports the H7b and H7c assumptions in the SEM model. By contrast, the results of analyzing the complex causal relationship of creativity, cooperativity, and algorithmic thinking for critical thinking showed that there is a pathway for the complex causal statement of creativity, cooperativity, algorithmic thinking, and critical thinking (consistency = 0.867, coverage = 0.760), which will lead to improved critical thinking ability. This supported the hypotheses of H8a, H8b, and H8c in the SEM model.

The results of further analysis of the complex causal relationship between empathic concern and critical thinking for improved CPS skills (Table 7) showed that high empathic concern and critical thinking (consistency = 0.890, coverage = 0.550) will lead to improved CPS skills. This supported the H7d and H8d assumptions in the SEM model.

## Discussion and conclusion

### Theoretical implication

To improve its ability to deal with complex practical problems, education has been committed to providing teaching measures that can stimulate subjects’ rational and irrational thinking. Healthcare professionals who utilize reflective learning must apply empathetic concern and critical thinking to confront challenges with high-quality solutions. Although previous studies confirmed the positive effects of empathic concern [19], and critical thinking [17] on CPS skills through symmetrical analysis, few studies have tested empathic concern and critical thinking at the same time. There is a dearth of studies that specifically investigate the factors that affect CPS skills in the context of reflective learning. And the previous studies on the factors influencing CPS skills mainly adopted traditional symmetric methods (such as regression and SEM), which did not fully capture the complexity behind the factors of influencing CPS skills. For instance, Hwang discovered that collaboration plays a crucial role in problem-solving, whereas communication may not be essential. In contrast, Kocak holds a contrasting perspective [21, 105]. The factors affecting the CPS skills are often based on multiple causalities rather than a single causal relationship. Therefore, based on the theory of multi-dimensional empathy [38] and 21st-century skills [21], we analyzed the data of 136 medical students undergoing reflective learning using PLS-SEM and fsQCA, and constructed a hypothetical model to examine the relationships between the CPS skills and influence factors (critical thinking, cooperativity, creativity, algorithmic thinking, empathic concern, fantasy, perspective-taking, personal distress).

The PLS-SEM results (Table 3) showed that a variety of attributes can affect the CPS skills, among which critical thinking and empathic concern play an intermediary role between most antecedents and CPS skills. The fsQCA results partly verified the mediating effect of critical thinking and empathetic concern (Tables 5 and 6). In the PLS-SEM results (Table 3), personal distress was identified to directly affect the CPS skills; and the effect of personal distress on empathic concern was not shown in the fsQCA solution (Table 5), which proved that personal distress directly affects the CPS skills without the intermediary of empathetic concern. This result is similar to that of Jeon [106]; however, he believed that there is a negative correlation between personal distress and problem solving, which might be related to the different learning patterns (reflective learning) used in this study. Personal distress is a necessary process in reflective learning because the motivation of prosocial behavior eases our uncomfortable state of mind by reducing the disgusting and awakening cues sent out by others [107]. Reflection urges us to face these emotions and draw lessons from them. Hoffman noted that excessive personal distress can turn others-oriented motivation into self-directed motivation, thus reducing the occurrence of prosocial behavior [54], which emphasizes the differential treatment of personal distress in different learning modes. In addition, perspective-taking was identified to affect the CPS skills directly (Table 3; C1 in Table 4) and indirectly (Table 3; C1 in Table 5). Therefore, some of the results obtained from fSQCA validated the conclusions of PLS-SEM to some extent (Table 8).

**Table 8:** Comparison between the results of PLS-SEM and fsQCA

	Methods	
Hypothesis	SEM	fsQCA
H1	Support	Conditional support
H2	Not supported	n.a. ^a^
H3	Support	n.a. ^a^
H4	Not supported	n.a. ^a^
H5	Not supported	n.a. ^a^
H6	Not supported	n.a. ^a^
H7a	Not supported	n.a. ^a^
H7b	Support	Conditional support
H7c	Support	Conditional support
H7d	Support	Conditional support
H8a	Support	Conditional support
H8b	Support	Conditional support
H8c	Support	Conditional support
H8d	Support	Conditional support

**Note**: ^a^ Not applicable

The fsQCA results provided more configuration solutions of complex causality, which extended the results of PLS-SEM and further revealed the complexity of affecting the CPS skills. For example, the fsQCA results in Table 7 not only proved the mediating effect of empathetic concern and critical thinking, but also suggest that they work together to affect the CPS skills. This demonstrates that CPS skills are impacted by both rational and irrational thinking, and positive emotions play a critical role in fostering CPS skills [108]. In Table 4 cooperativity, creativity, algorithmic thinking, critical thinking, and personal distress all appear in forward solutions with high coverage (C1, C2, C3 in Table 4). This suggested that personal distress, cooperativity, creativity, algorithmic thinking, and critical thinking can be regarded as the core conditions to affect the CPS skills, and these conditions make an important contribution in the context of reflective learning. Researches by Chen [77], Garrett [75], Geisinger [70] and Ellis [17] respectively believed that collaboration, creativity, algorithmic thinking and critical thinking play an important role in CPS skills, and Sze [109] believed that personal distress could have a positive impact on prosocial behavior and altruism. The studies above provide are similar to our perspective.

Critical thinking is not only one of the basic skills in the 21st Century but also a key ability in reflective learning [110, 111]. The process of questioning and reorganizing critical thinking is key to reflective learning. The complex structure of the problem-solving process requires critical thinking skills to find different solutions [30, 112–114]. Table 4 higher coverage solutions (C1, C2, C3, NC1, NC2, NC3 in Table 4) and Table 7 higher coverage solutions (C1, NC1 in Table 7) showed that critical thinking ability training is helpful to improve a subject’s CPS skills. By contrast, a lack of critical thinking training is not conducive to improving CPS skills (NC1, NC2, NC3 in Table 4; NC1 in Table 7). Critical thinking is widely considered to be a competency closely linked to CPS skills [29], and our study approves this perspective. Moreover, cooperativity, creativity, and algorithmic thinking also appear in the forward solutions with high coverage (C1, C2, C3 in Table 4), combined with the mediating effect of critical thinking on cooperativity, creativity, algorithmic thinking, and the CPS skills. It is logical that the antecedents of critical thinking, such as cooperativity, creativity and algorithmic thinking, also play a positive role in the CPS skills. The result is similar to the findings of Özgenel [115], who believed that critical thinking and creative thinking affected problem-solving skill through decision-making style. These results suggested that we should pay attention to the cultivation of a critical thinking ability, especially through the cultivation of cooperativity, creativity, and algorithmic thinking, which positively and significantly improve a subject’s ability to solve complex problems.

Empathetic concern relationship with the complex configuration between its antecedent variables provides new ideas and insights to improve our ability to solve complex problems. Empathic concern, as a key factor of prosocial behavior [43], is also of positive significance to CPS skills in this study. The higher coverage solutions (C1, C2, C3 in Table 4; NC1, NC2, NC3 in Table 4; C1 in Table 7; NC1 in Table 7) showed that training in empathic concern is beneficial to improve a subject’s CPS skills, while a lack of empathic concern training is not conducive to improving CPS skills (NC1, NC2 in Table 4; NC1 in Table 7). By contrast, fantasy (C2, C3 in Table 4) and perspective-taking (C1, C3 in Table 4) appeared among the forward solutions with higher coverage in Table 4. Combined with the mediating effect among empathic concern, fantasy, perspective-taking, and the CPS skills, it is not difficult for us to understand that the empathic concern antecedent variable: fantasy, and perspective-taking, also have positive significance for the CPS skills. This result aligns with the research findings of Hashmi [57] and Davenport [66]. Moreover, in the absence of empathetic concern, the pathway support of the combination of fantasy, perspective-taking, and personal distress for CPS skills also verified this positive significance from the other side (C3 in Table 4). However, personal distress (C1, C2, C3 in Table 4) appears independently in the forward solution, with high coverage in Table 4, which verifies the direct effect of personal distress on the CPS skills, which was consistent with the results of PLSSEM. These further confirmed the theory of Dorner and Funke, who suggested that complex and dynamic non-routine situations across different domains require a collection of self-regulating psychological processes and a creative combination of knowledge and strategies, and is influenced by motivation and emotion, especially in a high-stakes environment[24]. In addition, according to the observation of the reverse solution of Table 4, the combination of negative perspectivetaking and negative personal distress will be conducive to the low-level CPS skills (NC1, NC2, NC3, NC5 in Table 4). Interestingly, fantasy appeared not only in the forward solution with high coverage (C2, C3 in Table 4), but also in the inverse solution with high coverage (NC2, NC3 in Table 4), which seemed to suggest that the contribution of fantasy to improving CPS skills is neutral, which requires further research.

Consistent with the principle of causal asymmetry, fsQCA suggested that solutions generated by the same attributes in different areas might have the opposite impact on CPS skills, depending on how they combine or interact with other attributes. The lack or negation of some positive factors will lead to improved CPS skills, while the existence of some negative factors might also lead to similar results, depending on how they are configured with the other factors. For example, solution 4 in Table 4 shows that in the absence of critical thinking, cooperativity, algorithmic thinking, creativity, and fantasy, a combination of empathetic concern, personal distress, and perspective-taking could also have a positive effect on the improvement of CPS skills. There is a paucity of literature exploring the effects of empathetic concern, personal distress, and perspective-taking on CPS skills under conditions of low levels of critical thinking, cooperativity, algorithmic thinking, creativity, and fantasy. These insights provide new ideas for exploring improvements in CPS skills. Although PLS-SEM can verify the predetermined relationship between previous factors and the results of interest, it cannot provide these insights.

In addition, considering the complex nature of problem-solving skill under the condition of reflective learning, it is necessary to check the linear and nonlinear relationships between structures to fully understand the strategies and methods to improve CPS skills. In this study, as an ideal approach, PLS-SEM was used to identify the linear (symmetric) causal relationship between the improvement of CPS skills and influence factors. The fSQCA was used to identify the nonlinear (asymmetric), heterogeneous, and dynamic interactions between antecedents and behavioral results. The fSQCA improved identifying sufficient causal conditions for outcomes. The comprehensive application of PLS-SEM and fsQCA helped capture complex multiple causalities in the improvement of CPS skills, which makes theoretical contributions in terms of analytical techniques.

### Practical implications

Aquino believes that the implementation of reflective learning strategies is conducive to the improvement of CPS skills [116], which is of practical significance for the design of learning strategies and training tools, including reflective learning. The PLS-SEM results showed that perspective-taking, as an important condition for affected CPS skills, not only plays a role through the intermediary effect of empathetic concern, but also directly affects the CPS skills. The researchers and learners can train subjects to think for others in the form of team communication and exchange of views. For the sake of others, it is necessary to think about CPS skills solutions from multiple angles and more comprehensively, by thinking about problems from the standpoint and perspective of others. Therefore, it is necessary to adopt evidence-based strategies for training to improve the CPS skills.

While the fsQCA results confirmed the PLS-SEM results, in turn its complex configuration helps researchers and learners to make more informed decisions about learning methods to improve CPS skills. The derived pathways indicated that there is more than one causal configuration that can improve CPS skills, and how to improve depends on a combination of attributes. For example, our results showed that the high level of critical thinking and its antecedent attributes, combined with the high level of empathic concern, personal distress, and perspective-taking, will lead to improvement of the CPS skills. The lack of critical thinking, cooperativity, algorithmic thinking, creativity, and fantasy, which to some extent emphasizes the utility of empathic concern, personal distress, and perspective-taking (Table 4 solution 4), make it necessary to pay attention to training medical students in empathic concern, personal distress, and perspective-taking using reflective learning. Aligned with our own research, it was acknowledged reflective learning as a potent method to enhance empathy [37]. Medical reflection should focus on cultivating the ability to speculate on materials and self-views, and at the same time, understand decision-making from the situation of others and feel the emotions of others to trigger empathy. Improvement of CPS skills should not only emphasize the reduction of personal distress, but also should look at the role of personal distress critically. At the same time, it also reminds us that we should fully consider the training situation of the subjects in the design of learning strategies. Critical thinking and its antecedents are regarded as the key solutions in fsQCA, which suggests that we can focus on the reflective learning mode when we train subjects for critical thinking, creativity, cooperativity, and algorithmic thinking. We should also consciously use this kind of thinking to solve problems in the process of reflective learning. In the design of other learning strategies, training in critical thinking ability and its antecedent variables, cooperativity, creativity, and algorithmic thinking, can effectively help subjects to improve their CPS skills.

Based on our understanding of how empathic concern and critical thinking work together to improve the CPS skills, we suggest that real and complex problems in life be taken as examples in the choice of reflective teaching strategies, to involve a series of related skills and characteristics, and fully exercise the two modes of thinking. This is because, in reflective learning, subjects internalize the thinking skills taught by others into their own thinking skills, cultivating the ability to monitor and reflect on the whole problem-solving process, and helping subjects to extract useful strategies, experiences, and patterns into their cognitive structure, thereby improving their CPS skills and accumulating more experience for possible intuitive thinking. This is more suitable for problems based on real-life, which is in line with the medical learning problem-based learning and case-based learning models.

### Conclusion

In this study, a hypothetical model of the relationship between the CPS skills and influencing factors (critical thinking, cooperation, creativity, algorithmic thinking, empathic concern, fantasy, perspective-taking, and personal distress) was constructed and validated. The model confirmed the mediating effect of critical thinking and empathic concern on the CPS skills, the direct effect of personal distress, and the direct and indirect effect of perspective-taking on the CPS skills. Besides, fsQCA results provided a variety of configurations that enhanced the improvement of CPS skills. The findings not only enriched the theoretical system of affecting CPS skills, but also provided practical guidance for the development of learning strategies and assessment tools aimed at improving CPS skills.

## Limitations and future research

Although this study enriches the theoretical and practical knowledge concerning the relationship between CPS skills and critical thinking, empathic concern, and other variables, it also has some limitations. First, the subjects were beginners in terms of reflective learning under the guidance of teachers, and lack experience in reflective learning, which might affect the accuracy and applicability of variables to some extent. In future research, we will improve these shortcomings, practice reflective learning practices in more subjects, and validate the model in a broader learning strategy, which would be very meaningful. Second, based on the model of this study, it is necessary to enrich the paths and develop a variety of learning and training tools to improve the CPS skills in the future research. The development of assessment tools for factors related to the measurement of CPS skills will facilitate targeted training and realize personalized learning practice guidance.

## Electronic supplementary material

Below is the link to the electronic supplementary material.

Supplementary Material 1

## Data Availability

The data sets used and / or analyzed in this study have not been made public. If there is a reasonable need, they can be obtained from and provided by the corresponding author of this article.

## Abbreviations

CPS: Complex problem-solving
OECD: Organisation for Economic Co-operation and Development
PLS-SEM: Partial least square structural equation modeling
fsQCA: Fuzzy set qualitative comparative analysis
AVE: Average variance extracted
VIF: Variance inflation factor
IRI: Interpersonal Reactivity Index
CTS: Computational Thinking Scale
SEM: Structural equation modeling.
