# Supplementary material for: Factors influencing the complex problem-solving skills in reflective learning: results from partial least square structural equation modeling and fuzzy set qualitative comparative analysis
Source: BMC Med Educ. 2023 May 25;23:382. doi: 10.1186/s12909-023-04326-w (PMC10210286; doi:10.1186/s12909-023-04326-w)
Supplement: Supplementary file 1 — Supplementary Material 1 [file 12909_2023_4326_MOESM1_ESM.docx]

**Additional file. Supplementary materials**

|  | Fantasy | Personal Distress | Perspective-Taking | Empathic Concern | Algorithmic Thinking | Cooperativity | Creativity | Critical Thinking | CPS skills |
| --- | --- | --- | --- | --- | --- | --- | --- | --- | --- |
| Fantasy |  |  |  | 1.014 |  |  |  |  |  |
| Personal Distress |  |  |  |  |  |  |  |  | 1.138 |
| Perspective-Taking |  |  |  | 1.014 |  |  |  |  | 1.615 |
| Empathic Concern |  |  |  |  |  |  |  |  | 1.583 |
| Algorithmic Thinking |  |  |  |  |  |  | 1.587 |  |  |
| Cooperativity |  |  |  |  |  |  | 1.570 |  |  |
| Creativity |  |  |  |  |  |  | 1.498 |  |  |
| Critical Thinking |  |  |  |  |  |  |  |  | 1.051 |
| CPS skills |  |  |  |  |  |  |  |  |  |

**Additional file. 1** Inner VIF values.

**Additional file. 2** Necessary solutions of CPS skills.

| Outcome variable: CPS skills | | |
| --- | --- | --- |
| Conditions tested: | | |
|  | Consistency | Coverage |
| Fantasy | 0.707 | 0.736 |
| Perspective-taking | 0.970 | 0.601 |
| Personal Distress | 0.731 | 0.729 |
| Empathic Concern | 0.789 | 0.800 |
| Cooperativity | 0.740 | 0.695 |
| Creativity | 0.712 | 0.714 |
| Algorithmic Thinking | 0.699 | 0.743 |
| Critical Thinking | 0.698 | 0.736 |
|  |  |  |
| Outcome variable: ~ CPS skills | | |
| Conditions tested: |  |  |
|  | Consistency | Coverage |
| Fantasy | 0.620 | 0.634 |
| Perspective-taking | 0.974 | 0.593 |
| Personal Distress | 0.608 | 0.597 |
| Empathic Concern | 0.548 | 0.546 |
| Cooperativity | 0.647 | 0.598 |
| Creativity | 0.644 | 0.635 |
| Algorithmic Thinking | 0.631 | 0.659 |
| Critical Thinking | 0.581 | 0.602 |

**Additional file. 3** Necessary solutions for Empathic Concern.

| Outcome variable: Empathic Concern | | |
| --- | --- | --- |
| Conditions tested: | | |
|  | Consistency | Coverage |
| Fantasy | 0.754 | 0.690 |
| Perspective-taking | 0.780 | 0.726 |
| Personal Distress | 0.651 | 0.644 |
|  |  |  |
| Outcome variable: ~Empathic Concern | | |
| Conditions tested: |  |  |
|  | Consistency | Coverage |
| Fantasy | 0.604 | 0.623 |
| Perspective-taking | 0.576 | 0.605 |
| Personal Distress | 0.578 | 0.644 |

**Additional file. 4** Necessary solutions of Critical thinking.

| Outcome variable: Critical Thinking | | |
| --- | --- | --- |
| Conditions tested: | | |
|  | Consistency | Coverage |
| Cooperativity | 0.929 | 0.694 |
| Creativity | 0.834 | 0.740 |
| Algorithmic Thinking | 0.920 | 0.710 |
|  |  |  |
| Outcome variable: ~Critical Thinking | | |
| Conditions tested: |  |  |
|  | Consistency | Coverage |
| Cooperativity | 0.647 | 0.607 |
| Creativity | 0.546 | 0.607 |
| Algorithmic Thinking | 0.623 | 0.604 |
